# Motor experience with a sport-specific implement affects motor imagery

**DOI:** 10.7717/peerj.4687

**Published:** 2018-04-27

**Authors:** Lanlan Zhang, Yanling Pi, Hua Zhu, Cheng Shen, Jian Zhang, Yin Wu

**Affiliations:** 1 School of Kinesiology, Shanghai University of Sport, Shanghai, China; 2 Shanghai Punan Hospital of Pudong New District, Shanghai, China; 3 School of Economics and Management, Shanghai University of Sport, Shanghai, China

**Keywords:** Motor imagery, Temporal congruence, Implement, Facilitation, Interference

## Abstract

The present study tested whether sport-specific implements facilitate motor imagery, whereas nonspecific implements disrupt motor imagery. We asked a group of basketball players (experts) and a group of healthy controls (novices) to physically perform (motor execution) and mentally simulate (motor imagery) basketball throws. Subjects produced motor imagery when they were holding a basketball, a volleyball, or nothing. Motor imagery performance was measured by temporal congruence, which is the correspondence between imagery and execution times estimated as (imagery time minus execution time) divided by (imagery time plus execution time), as well as the vividness of motor imagery. Results showed that experts produced greater temporal congruence and vividness of kinesthetic imagery while holding a basketball compared to when they were holding nothing, suggesting a facilitation effect from sport-specific implements. In contrast, experts produced lower temporal congruence and vividness of kinesthetic imagery while holding a volleyball compared to when they were holding nothing, suggesting the interference effect of nonspecific implements. Furthermore, we found a negative correlation between temporal congruence and the vividness of kinesthetic imagery in experts while holding a basketball. On the contrary, the implement manipulation did not modulate the temporal congruence of novices. Our findings suggest that motor representation in experts is built on motor experience associated with specific-implement use and thus was subjected to modulation of the implement held. We conclude that sport-specific implements facilitate motor imagery, whereas nonspecific implements could disrupt motor representation in experts.

## Introduction

Motor imagery is defined as the mental representation of movement with no concomitant production of muscular activity (Jeannerod, 2001). Motor imagery has functional equivalence to motor execution such that the two processes share partially overlapping neural substrates (Jeannerod, 1994; Grezes & Decety, 2001; Holmes & Collins, 2001; Grèzes et al., 2003). Motor imagery has been suggested to be effective in improving motor performance (Jackson et al., 2003; Mulder et al., 2004; Allami et al., 2008; Mizuguchi et al., 2012; Schack et al., 2014).

Temporal congruence is the correspondence between the time of imagined movements and that of actual movements which reflects the individual ability to preserve the temporal organization of the actual performance during motor imagery (Guillot & Collet, 2005; O & Hall, 2009; Guillot et al., 2012). The difficulty in achieving temporal congruence has been taken as imagery impairment (Sirigu et al., 1996; Malouin et al., 2004; Guillot et al., 2012) and associated with a worse performance improvement following motor imagery training (Holmes & Collins, 2001; Guillot & Collet, 2005; Guillot & Collet, 2008; Louis et al., 2008; Malouin et al., 2008). Temporal congruence is affected by a series of factors including imagery speed (Decety & Jeannerod, 1995; O & Hall, 2009; Guillot et al., 2012), expertise level (Reed, 2002; Louis, Collet & Guillot, 2011; Guillot et al., 2012), environmental context (Holmes & Collins, 2001; Guillot, Collet & Dittmar, 2005; Guillot & Collet, 2008), and so forth.

The acquirement of motor skills in most sports is accompanied with the use of sport-specific implements. The specific implement is one of the fundamental but dispensable “basic action concepts” for building and optimizing mental representations of complex sport movements to obtain promising mental practice efficacy (Schack, 2004; Schack et al., 2014). Motor experience with a sport-specific implement has been suggested to influence motor representation of complex sport movement (Guillot, Collet & Dittmar, 2005; Fourkas et al., 2008; Bisio et al., 2014; Wang et al., 2014). Our previous neurophysiological study revealed increased corticospinal excitability in experts during motor imagery of badminton serving while holding a badminton racket compared to that while holding a plastic bar (Wang et al., 2014), suggesting that specific implement induced better motor imagery performance compared to nonspecific implement. However, due to the absence of a holding nothing condition, whether the difference resulted from a facilitation effect from the sport-specific implement or an interference effect from a nonspecific implement is unclear. In other words, comparing the performance of motor imagery with the sport-specific implement, to that of when holding nothing enables us to examine the potential facilitation effect of the specific implement; comparing the performance of motor imagery with a nonspecific implement, to that of when holding nothing enables us to examine the potential interference effect of a nonspecific implement.

To address these considerations, we asked a group of experienced basketball players and a group of novices to perform motor execution and motor imagery of basketball throw. Basketball throw was utilized as representative of complex sport movements. Subjects produced motor imagery in three experimental conditions: holding a basketball (HB, sport-specific implement), holding a volleyball (HV, nonspecific implement), or holding nothing (HN). To assess the performance of motor imagery, the temporal congruence between motor execution and motor imagery as well as the vividness of motor imagery were evaluated. We examined the potential facilitation effect from the sport-specific implement by comparing motor imagery performance of basketball throw when subjects held a basketball (HB) to that of when they held nothing (HN); we examined the potential interference effect from a nonspecific implement by comparing motor imagery performance of basketball throw when subjects held a volleyball (HV) to that of when they held nothing (HN).

## Materials & Methods

### Subjects

Twenty-four basketball players (mean age ± standard deviation [*SD*] = 19.7 ±  1.6 years, age range 18–23 years), and 24 age-matched novices (healthy controls, mean age ± *SD* = 20.1 ± 1.5 years, age range 17–23 years) were studied. All subjects were identified as right-handed males (Oldfield, 1971). Elite players were national first and second level players who competed frequently at the national and international levels. They trained on average 10.7 ± 1.7 h (mean ± *SD*) per week for 10.8 ± 1.9 years (mean ± *SD*, ranged 8–14 years). Novices were university students who had no experience in professional training in basketball or any other sports. The experimental procedure was approved by the local ethics committee at the Shanghai University of Sport (No.2017106) and all subjects gave written informed consent prior to the experiment.

### Mental chronometry test

**Figure 1:**
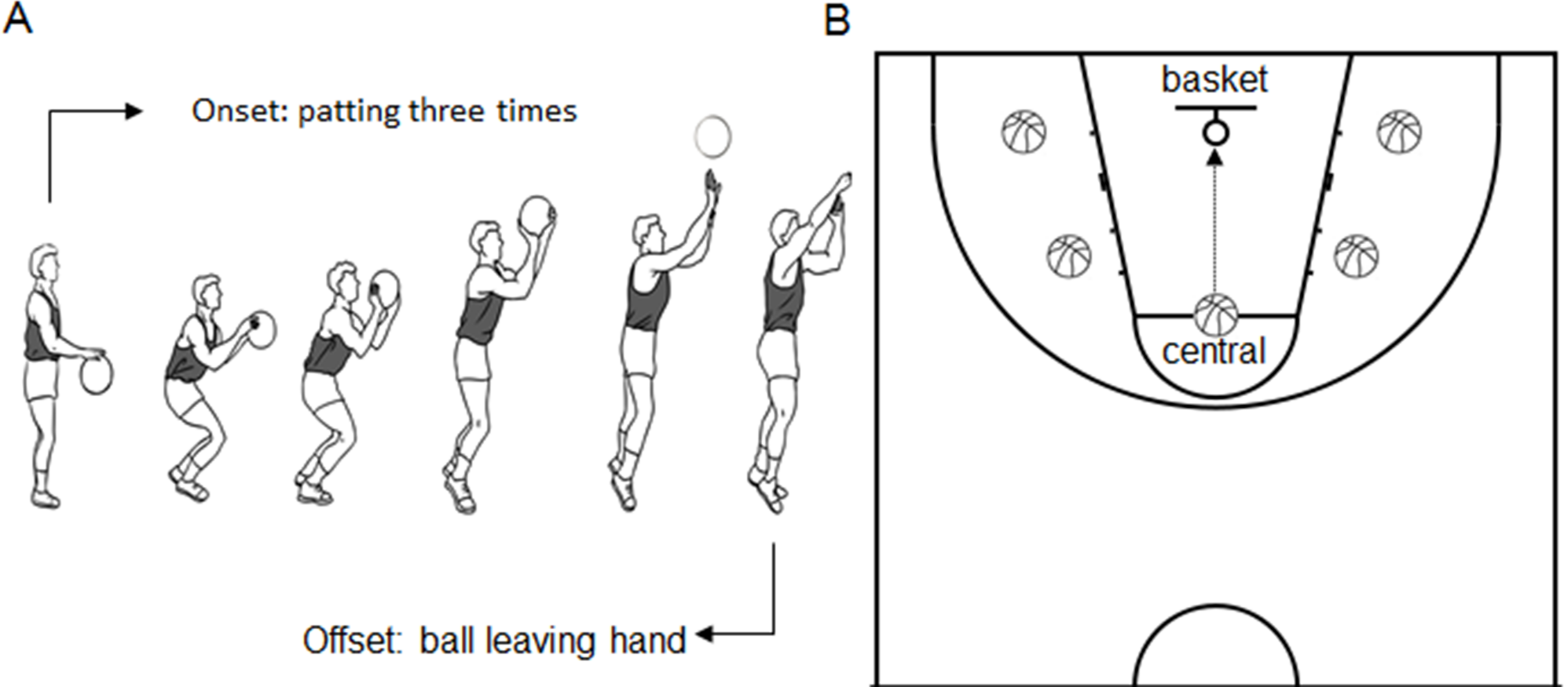
Experimental design. (A) The decomposed movements for the basketball free throw task. The onset movement is ball dribbling and the offset is the ball leaving the hand. The time for basketball free throw is estimated as the duration between onset and offset. (B) Five locations (labeled with balls) for basketball free throw. The center location is the traditional location for a free throw. The other four locations have the same distance to the basket as the center location.

Basketball throw (shooting the *basketball* toward the basket after dribbling it in place three times) was used as the motor task (Fig. 1A). Five locations on the court were prescribed for basketball throw (Fig. 1B). The central location was at the traditional point for free throw at the center. The other four lateral locations were located bilaterally to the first location. All five locations had the same distance to the basket. The location setting is intended to avoid possible fatigue or adaptation effect during motor execution or imagery from a single location. Field training was applied to all subjects before the experiment to help subjects understand the motor task used in the present study.

Motor execution. Subjects physically performed basketball throws on the court from each of the 5 locations. The duration of motor execution was defined as the time between the onset of the first dribble and the offset when the ball left the subject’s hand. Experimenters recorded the duration of each trial with a stopwatch. Three trials were repeated at each of the 5 locations (total 15 trials). The duration of motor execution for all 15 trials was averaged and the mean value was defined as execution time.

Motor imagery. Subjects mentally rehearsed basketball throws, that is, dribbling and shooting a *basketball*, while holding a basketball (HB), a volleyball (HV) or nothing (HN) from each of the 5 locations on the court. Subjects were instructed to mentally rehearse basketball throws with their eyes closed in a first-person perspective during motor imagery (Wang et al., 2014) and in real-time speed as they did during motor execution (O & Hall, 2009). We measured the motor imagery time of basketball throws in the two groups when subjects held a basketball (HB), a volleyball (HV) and held nothing (HN). Three trials at each of the 5 locations (total 15 trials) were repeated for both the with-ball and without-ball conditions. In each trial, subjects verbally indicated the onset and offset of motor imagery by saying “start” and “stop” and the experimenter monitored durations with a stopwatch (Bisio et al., 2014). The duration of motor imagery of the 15 trials each for with-basketball, with-volleyball and without-ball conditions were averaged separately and the mean values were defined as imagery time for the corresponding conditions.

Temporal congruence. To normalize the inter-individual differences, we calculated the “delta time” developed from the formula used in Meulen et al. (2014) to estimate temporal congruence: delta = (imagery time − execution time)/(imagery time + execution time). Therefore, the smaller the delta score, the greater the temporal congruence between motor execution and motor imagery. Temporal congruence was calculated for the three experimental conditions separately. Comparing the temporal congruence in different conditions thus revealed the effect of somatosensory input on motor imagery.

### Introspective reports

Immediately after subjects completed the motor imagery task, the quality of motor imagery was evaluated with introspective reports via two questionnaires. The first questionnaire tested the perspective subjects used for motor imagery and was developed from that used in Wang et al. (2014). The test includes 4 introspective questions with a 5-point scale (5 being the most positive). The first question asks the subjects whether they used first-person perspective during motor imagery; the second question asks the subjects whether the first-person perspective motor imagery was easily controlled; the third question asks whether the first-person perspective motor imagery was clear; the fourth question asks for the difference between ease to perform first-person perspective motor imagery in different experimental conditions. The second questionnaire tested the vividness of motor imagery and was developed from that used in Fourkas et al. (2008). The second questionnaire included 8 questions related to the kinesthetic and visual properties (4 for each) during the motor imagery on a 7-point scale with 7 representing the greatest vividness during imagery. The questions were adapted to be linked to the specificity of the experimental task and condition. Four kinesthetic questions were about difficulty of imagery, sequence of muscle contraction, muscle tension and throwing force during imagery. Four visual questions were about clarity of the court, clarity of the ball, clarity of the basket and clarity of the basketball throw movements. Note that the use of first-person perspective was estimated in overall regard while the vividness of motor imagery was rated under three experimental conditions (HB, HV and HN), separately.

### Data analysis

#### Temporal congruence

For temporal congruence, we carried out a two-way repeated measure analysis of variance (ANOVA), with group (2 levels, experts and novices) as a between-subject factor and the experimental condition (3 levels, HB, HV and HN) as a within-subject factor). We tested for differences among the three conditions by performing post hoc *t*-tests with Bonferroni correction.

#### Post-imagery questionnaires

The use of a first-person perspective during motor imagery was tested with unpaired *t*-tests. The vividness of motor imagery data combining kinesthetic and visual properties was also analyzed with a two-way repeated measures ANOVA in the same way as done for temporal congruence. To further examine the effect of kinesthetic imagery, the vividness of kinesthetic imagery was entered into a separate two-way repeated measures ANOVA.

#### Correlation analysis

As the distribution of vividness is not normal (for vividness of motor imagery, Kolmogorov–Smirnov = .18, *p* = .049; for vividness of kinesthetic imagery, Kolmogorov–Smirnov = .18, *p* = .055), nonparametric Spearman rank correlation was performed to explore the relationship between objective measure (mental chronometry) and subjective measure (vividness of motor/kinesthetic imagery) for experts during motor imagery in the with-basketball condition.

## Results

### Temporal congruence

**Figure 2:**
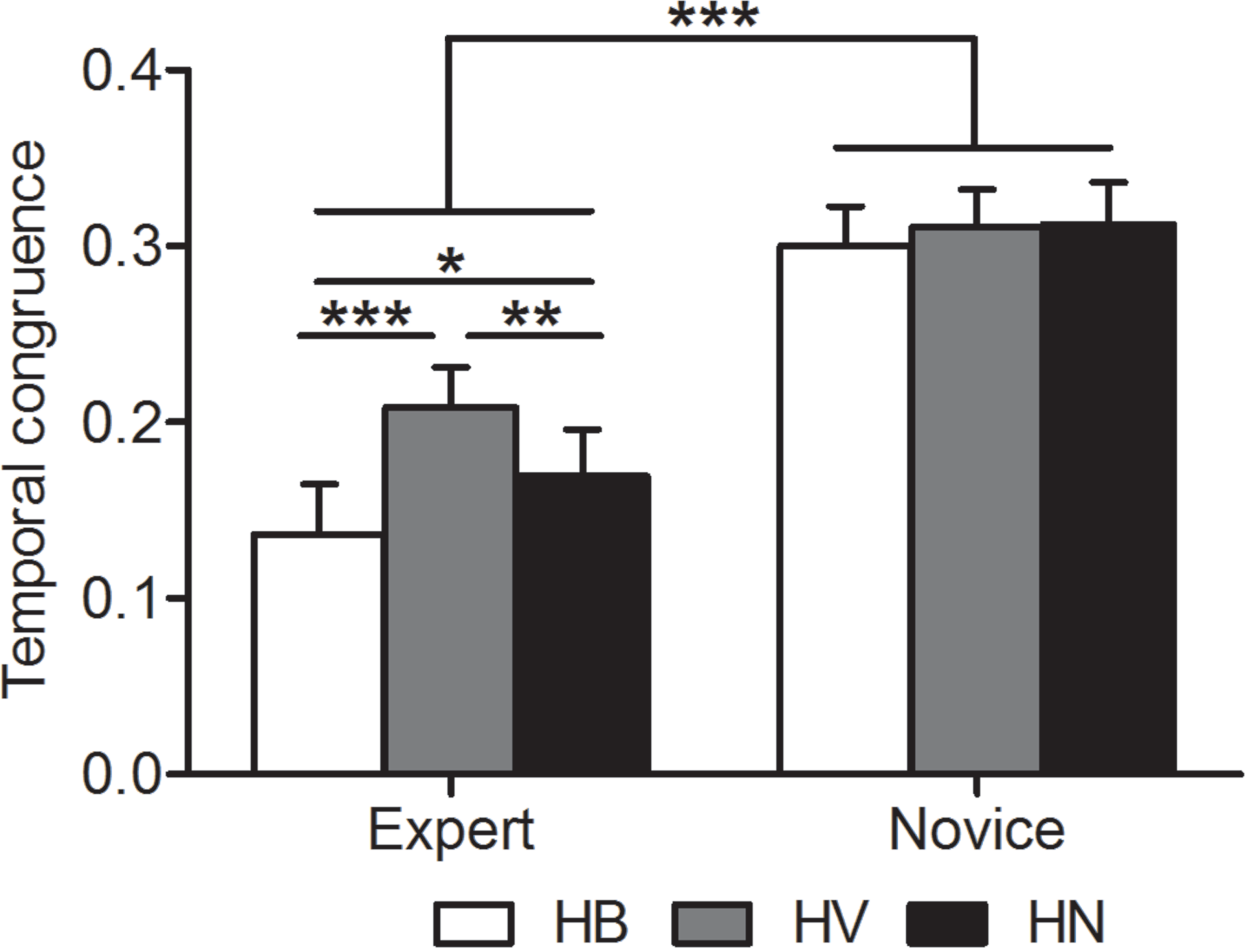
Temporal congruence. The ordinate shows temporal congruence. It is expressed as (imagery time) minus (execution time) divided by (imagery time plus execution time). The smaller the ratio score, the better the temporal congruence between motor execution and motor imagery. *, *p* < .05; **, *p* < .01; ***, *p* < .001. HB, holding a basketball; HV, holding a volleyball; HN, holding nothing. Error bars indicate standard error.

The duration of motor execution and motor imagery was listed in Table 1. Two-way repeated measures ANOVA on temporal congruence revealed significant main effects for group, *F*(1, 46) = 14.91, *p* < .0005, *η*^2^ = .25, and experimental condition, *F*(2, 92) = 12.08, *p* < .0005, *η*^2^ = .21, as well as an interaction effect between group and experimental condition, *F*(2, 92) = 6.89, *p* = .002, *η*^2^ = .13 (Fig. 2). Post hoc *t*-tests with Bonferroni correction confirmed that experts showed greater temporal congruence in all experimental conditions compared to novices (mean ± stand error [*SE*] for HB: experts, .14 ± .03, novices, .29 ± .02, *p* < .0005; HV: experts, .21 ± .02, novices: .3 ± .02, *p* = .004; HN: experts, .17 ± .03, novices, .3 ± .03, *p* < .0005). For experts, motor imagery with a basketball showed greater temporal congruence with motor execution than that with a volleyball (*p* < .0005) and that without a ball (*p* = .034); motor imagery without a ball showed greater temporal congruence with motor execution than that with a volleyball (*p* = .006). No significant difference was found among experimental conditions for novices (always *p* > .9).

**Table 1:** Mean ±  SE for motor execution time and motor imagery time.

Group	Motor execution	Motor imagery		
		HB	HV	HN
Expert	2.87 ± .08	3.95 ± .24	4.51 ± .23	4.19 ± .23
Novice	2.49 ± .10	4.61 ± .20	4.73 ± .22	4.80 ± .26

**Notes.**

SE standard error HB Holding a basketball HV Holding a volleyball HN Holding nothing

### Retrospective reports

Unpaired *t*-test using first-person perspective revealed no significant difference between experts and novices using first-perspective during motor imagery (mean ± SE for experts, 14.91 ± .51, novices, 14.38 ± .48, *t*(46) = .78, *p* = .44, Cohen’s *d* = .23).

**Figure 3:**
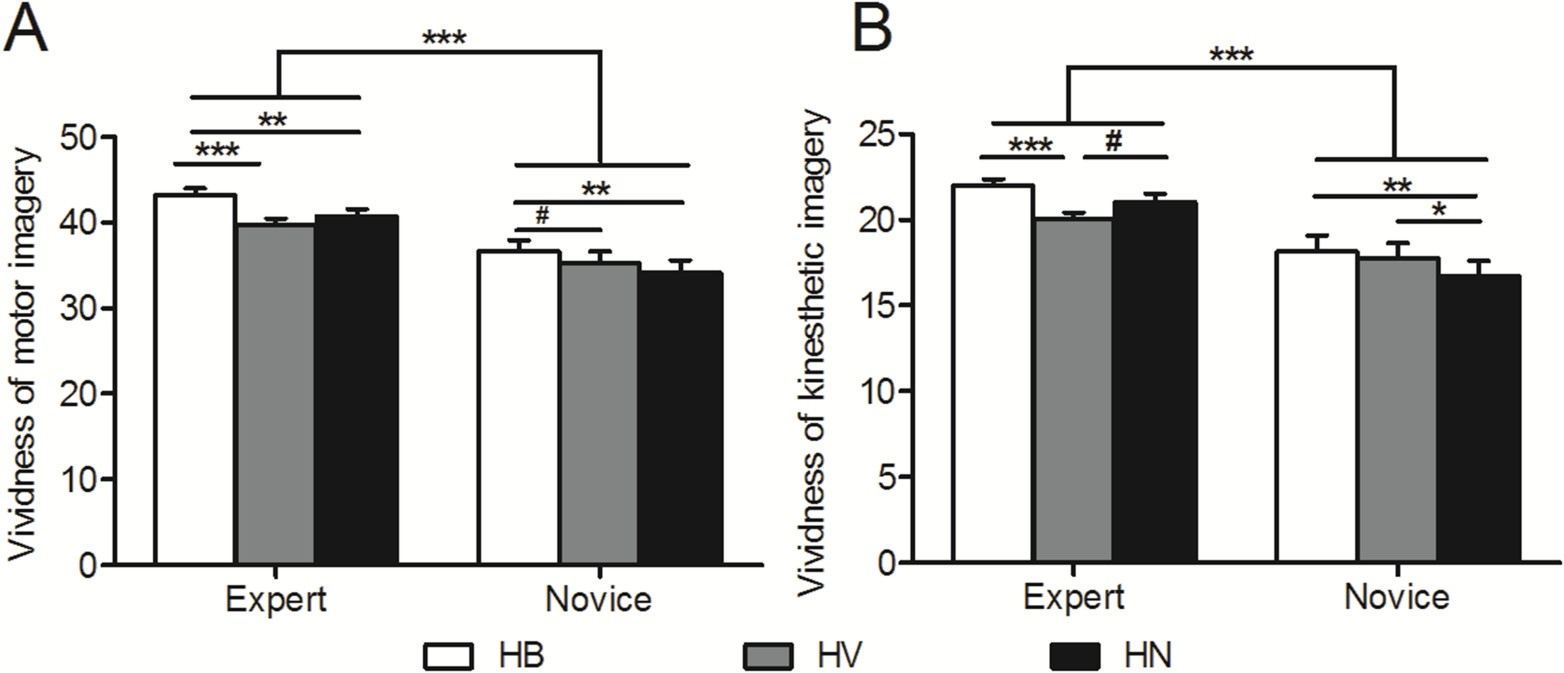
Subjective evaluation of motor imagery. (A) Vividness of motor imagery. Vividness of motor imagery is expressed as MIQ scores combining kinesthetic and visual properties. (B) Vividness of kinesthetic imagery. *, *p* < .05; **, *p* < .01; ***, *p* < .001; #, marginal significance. Error bars indicate standard error of the mean. (A) Vividness of motor imagery. Vividness of motor imagery combines kinesthetic and visual properties. (B) Vividness of kinesthetic imagery. *, *p* < .05; **, *p* < .01; ***, *p* < .001; #, marginal significance. HB, holding a basketball, HV, holding a volleyball, HN, holding nothing. Error bars indicate standard error.

The scores for vividness of motor imagery were listed in Table 2. Two-way repeated measures ANOVA on vividness of motor imagery combining kinesthetic and visual properties revealed significant main effects of group, *F*(1, 46) = 15.02, *p* < .0005, *η*^2^ = .25) and experimental condition, *F*(2, 92) = 25.88, *p* < .0005, *η*^2^ = .36), as well as an interaction effect between group and experimental condition, *F*(2, 92) = 4.69, *p* = .014, *η*^2^ = .09 (Fig. 3A). Post hoc *t*-tests with Bonferroni correction confirmed that experts showed greater vividness for motor imagery in all experimental conditions compared with novices (HB: *p* < .0005; HV: *p* = .008; HN: *p* < .0005). For experts, vividness for motor imagery with a basketball was higher compared to that with a volleyball (*p* < .0005) and that without a ball (*p* = .001). However, no significant differences were found between the other two experimental conditions (HV vs. HN, *p* = .12). For novices, vividness for motor imagery with a basketball was higher than that without a ball (*p* = .001) and the difference between vividness of motor imagery with a basketball and that with a volleyball tended to be significant (*p* = .051). No significant difference was found between vividness of motor imagery with a volleyball and that without a ball (*p* = .102).

**Table 2:** Mean ±  SE for vividness of motor imagery.

Group	HB	HV	HN
Expert			
Kinesthetic	22.00 ± .36	20.08 ± .35	21.04 ± .47
Visual	21.25 ± .53	19.67 ± .47	19.71 ± .47
Total	43.25 ± .74	39.75 ± .75	40.75 ± .83
Novice			
Kinesthetic	18.17 ± .92	17.75 ± .88	16.71 ± .91
Visual	18.46 ± .56	17.50 ± .66	17.50 ± .66
Total	36.63 ± 1.30	35.25 ± 1.43	34.20 ± 1.41

**Notes.**

SE standard error HB Holding a basketball HV Holding a volleyball HN Holding nothing

For kinesthetic imagery, two-way repeated measures ANOVA also revealed significant main effects of group, *F*(1, 46) = 14.18, *p* < .0005, *η*^2^ = .24, experimental condition, *F*(2, 92) = 11.36, *p* < .0005, *η*^2^ = .2, as well as an interaction effect between group and experimental condition, *F*(2, 92) = 6.54, *p* = .003, *η*^2^ = .13 (Fig. 3B). Post hoc *t*-tests with Bonferroni correction confirmed that experts showed higher vividness for kinesthetic imagery in all experimental conditions compared with novices (HB: *p* < .0005; HV: *p* = .017; HN: *p* < .0005). For experts, motor imagery with a basketball showed higher vividness compared to that with a volleyball (*p* < .0005), and motor imagery without a ball tended to show higher vividness compared to that with a volleyball (*p* = .06). However, no significant differences were found between vividness of kinesthetic imagery with a basketball and that without a ball (*p* = .11). For novices, motor imagery without a ball showed lower vividness compared to that with a basketball (*p* = .006) and that with a volleyball (*p* = .039). No significant difference was found between vividness of kinesthetic imagery with a basketball and that with a volleyball (*p* = .791).

### Correlation between vividness of motor imagery and temporal congruence

**Figure 4:**
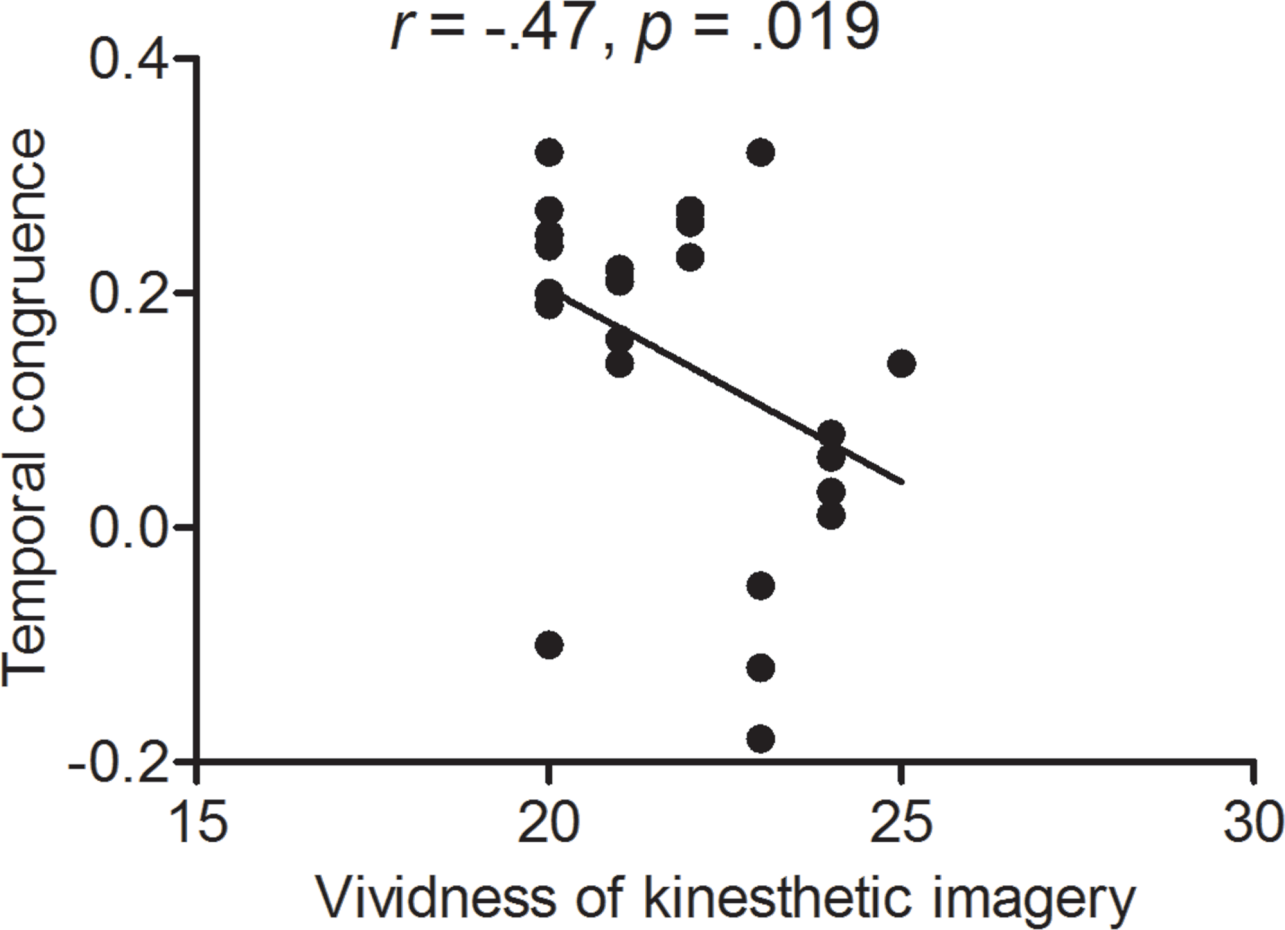
Correlation between subjective and objective measures in experts. The abscissa shows subjective measure as expressed by vividness of kinesthetic imagery and the ordinate shows objective measure as expressed by temporal congruence.

For experts during motor imagery while holding a basketball, no significant correlation was found between temporal congruence and vividness of motor imagery combining kinesthetic and visual properties, *r*(24) = .12, *p* = .593; however, a significant negative correlation between temporal congruence and kinesthetic imagery was found, *r*(24) =  − .47, *p* = .019), indicating that better temporal congruence was associated with more vivid imagery (Fig. 4).

## Discussion

The aim of the present study was to assess the potential facilitation effect of the sport-specific implement and the potential interference effect of a nonspecific implement on motor imagery of complex sport movements. Our main results show that experts obtained higher temporal congruence and, to a lesser degree, greater vividness of kinesthetic imagery when holding a sport-specific implement compared to that when holding nothing, and when holding nothing compared to when holding a nonspecific implement. These results demonstrate a facilitation effect for sport-specific implements and an interference effect for nonspecific implements on motor imagery.

### Prolonged duration of motor imagery

In all experimental conditions for both groups, the duration of motor imagery took longer than that of motor execution (Table 1). This is consistent with the findings of a series of previous studies (Bisio et al., 2014; Wang et al., 2014; Mizuguchi et al., 2015). During motor imagery, the temporal assembly of movement components is a more voluntary process that requires participants to not only pay attention to each movement component but also to feel the kinesthetic sensation of the movements. This could be true for both experts and novices. Specifically, for experts, sensory feedback is repressed during actual execution to allow for fluent, automatic motor performance, which could result in a shorter motor execution time (Oechslin et al., 2012). Novices showed even more prolonged duration of motor imagery than experts, which seems reasonable as (perceived) task difficulty or complexity is thought to influence the duration of mental representations of a movement (Guillot & Collet, 2005; Bakker et al., 2007). It is very likely that the basketball throw task appeared to be more difficult and complex for novices, taking it into consideration that novices had no basketball training experience.

### Influence of expertise level

We found that in all experimental conditions, the performance of motor imagery in experts was superior than that in novices, as suggested by greater temporal congruence (Fig. 2) and greater vividness of motor imagery (Fig. 3). The superior performance of motor imagery in experts is well acknowledged (Guillot & Collet, 2005; Louis, Collet & Guillot, 2011; Guillot et al., 2012; Bisio et al., 2014; Wang et al., 2014). As experts have gained high levels of automaticity from long-term repetitive training, they may be able to preserve the actual temporal organization of the movements. However, preserving the temporal feature is less likely to happen in novices as the lack of practice makes it unlikely for novices to encode the kinematic movement sequences into their motor repertoire. This is in line with the study of Bisio et al. (2014) who found more stereotyped motor pattern of forehand into the wall in tennis players than in tennis novices.

### Long-term utilization of a specific implement

Interestingly, the lowest temporal congruence and vividness of kinesthetic imagery were observed in experts while holding a volleyball (Fig. 2 and Fig. 3B). Thus, the significant differences between motor imagery performance associated to a basketball and that associated to no ball indicated a facilitation effect for the basketball, and the significant differences between motor imagery performance associated to a volleyball and that associated to no ball indicated an interference effect for volleyball. The facilitation effect from holding a basketball is not equivalent to the effect of a foam ball on imagery of squeezing movements (Mizuguchi et al., 2009; Mizuguchi et al., 2011), as holding a volleyball failed to induce similar effects. Unlike the simple squeezing movement with a foam ball, the maturation of basketball motor skill required long-term intensive practice. Therefore, it is likely that the facilitation effect from a basketball is associated to motor experience with the specific implement utilization during development of expertise skills. Similar findings related to the modulation of specific implement in experts were also found in studies on badminton (Wang et al., 2014), tennis (Fourkas et al., 2008; Bisio et al., 2014) and table tennis (Guillot, Collet & Dittmar, 2005). The underlying mechanism of interference effect from a nonspecific implement may come from divergence between central representation and peripheral input (Saimpont et al., 2012; Mizuguchi et al., 2015). It is likely that experts have built a well-defined mental representation dendrogram of sport-specific movements (Schack et al., 2012). Without modification, this well-established motor representation dendrogram of sport movements is specifically associated to a basketball and cannot be readily or effectively recruited by a volleyball.

The different experimental conditions failed to modulate temporal congruence in novices (Fig. 2). This was consistent with previous findings (Bisio et al., 2014). Our findings, together with evidence from previous studies (Bisio et al., 2014; Wang et al., 2014), suggest that it is necessary to have motor experience of using an implement to form its sensorimotor representation, and consequently to make a difference on motor imagery performance. However, novices did subjectively “feel” the motor imagery process to be more vivid when they were holding a ball, whether basketball or volleyball, compared to the feeling when they were holding nothing (Fig. 3B). We speculate this was because the tactile sensory input per se from a ball facilitated their mental image. Unlike experts, novices did not have the sport-specific motor experience associated to a basketball, thus the facilitation from tactile sensory input of a ball (regardless sport-specific or not) may be similar to that found in studies showing a facilitation of a foam ball on squeezing movements (Mizuguchi et al., 2009; Mizuguchi et al., 2011).

### Relationship between vividness of kinesthetic imagery and temporal congruence

We found that, for experts, greater vividness of kinesthetic imagery predicted higher temporal congruence when holding a basketball (Fig. 4). This finding is consistent with those of previous studies (Sirigu et al., 1996; Mizuguchi et al., 2015). Thus, it is likely that the compatibility between well-established internal representation built on motor experience of using a specific implement (basketball), and external somatosensory input facilitates the representation of basketball throws in experts.

### Limitations

The present study has limitations. Subjects were instructed to physically perform basketball throws only while holding a basketball but not while holding a volleyball or holding nothing. This design makes it unlikely to test the effect of somatosensory input on the imagery of pure kinematics of basketball throw movements. However, sport-specific implements such as rackets have been reported to feel like a hand extension (Fourkas et al., 2008; Bisio et al., 2014; Wang et al., 2014). Moreover, sport-specific implements have been taken as a fundamental unit for building mental movement representation during motor imagery trainings (Schack, 2004; Schack et al., 2014). Therefore, it is reasonable to test the effect of somatosensory input on the “kinematics + basketball” entity as the motor task was defined in the present study. Besides, the design in this study is not eligible to infer whether the observed effects from different implements on motor imagery apply to actual movements. Future study with a full design is expected to address this unknown.

## Conclusions

Previous studies demonstrated that motor representation of experts who developed motor skills associated to implement-use is reliant on the implement used to practice movements (Fourkas et al., 2008; Bisio et al., 2014; Wang et al., 2014). In line with the previous studies, the present study found sport-specific implement facilitates motor representation during motor imagery in experts but not novices. Further, the present study revealed interference effect of nonspecific implement on motor representation in experts during motor imagery.

##  Supplemental Information

10.7717/peerj.4687/supp-1 Data S1 Raw data for all measures were well-organized

## Additional Information and Declarations

### Data Availability

The following information was supplied regarding data availability: The raw data are provided in Data S1.
